# Daily Intravoxel Incoherent Motion (IVIM) In Prostate Cancer Patients During MR-Guided Radiotherapy—A Multicenter Study

**DOI:** 10.3389/fonc.2021.705964

**Published:** 2021-08-13

**Authors:** Ernst S. Kooreman, Petra J. van Houdt, Rick Keesman, Vivian W. J. van Pelt, Marlies E. Nowee, Floris Pos, Karolina Sikorska, Andreas Wetscherek, Arndt-Christian Müller, Daniela Thorwarth, Alison C. Tree, Uulke A. van der Heide

**Affiliations:** ^1^Department of Radiation Oncology, The Netherlands Cancer Institute, Amsterdam, Netherlands; ^2^Department of Biometrics, The Netherlands Cancer Institute, Amsterdam, Netherlands; ^3^Joint Department of Physics, The Royal Marsden NHS Foundation Trust and The Institute of Cancer Research, London, United Kingdom; ^4^Department of Radiation Oncology, University of Tübingen, Tübingen, Germany; ^5^Section of Biomedical Physics, Department of Radiation Oncology, University of Tübingen, Tübingen, Germany

**Keywords:** MR-linac, intravoxel incoherent motion, quantitative MRI, prostate cancer, treatment response

## Abstract

**Purpose:**

Daily quantitative MR imaging during radiotherapy of cancer patients has become feasible with MRI systems integrated with linear accelerators (MR-linacs). Quantitative images could be used for treatment response monitoring. With intravoxel incoherent motion (IVIM) MRI, it is possible to acquire perfusion information without the use of contrast agents. In this multicenter study, daily IVIM measurements were performed in prostate cancer patients to identify changes that potentially reflect response to treatment.

**Materials and Methods:**

Forty-three patients were included, treated with 20 fractions of 3 Gy on a 1.5 T MR-linac. IVIM measurements were performed on each treatment day. The diffusion coefficient (D), perfusion fraction (f), and pseudo-diffusion coefficient (D*) were calculated based on the median signal intensities in the non-cancerous prostate and the tumor. Repeatability coefficients (RCs) were determined based on the first two treatment fractions. Separate linear mixed-effects models were constructed for the three IVIM parameters.

**Results:**

In total, 726 fractions were analyzed. Pre-treatment average values, measured on the first fraction before irradiation, were 1.46 × 10^−3^ mm^2^/s, 0.086, and 28.7 × 10^−3^ mm^2^/s in the non-cancerous prostate and 1.19 × 10^−3^ mm^2^/s, 0.088, and 28.9 × 10^−3^ mm^2^/s in the tumor, for D, f, and D*, respectively. The repeatability coefficients for D, f, and D* in the non-cancerous prostate were 0.09 × 10^−3^ mm^2^/s, 0.05, and 15.3 × 10^−3^ mm^2^/s. In the tumor, these values were 0.44 × 10^−3^ mm^2^/s, 0.16, and 76.4 × 10^−3^ mm^2^/s. The mixed effects analysis showed an increase in D of the tumors over the course of treatment, while remaining stable in the non-cancerous prostate. The f and D* increased in both the non-cancerous prostate and tumor.

**Conclusions:**

It is feasible to perform daily IVIM measurements on an MR-linac system. Although the repeatability coefficients were high, changes in IVIM perfusion parameters were measured on a group level, indicating that IVIM has potential for measuring treatment response.

## Introduction

Integrated MR-linac systems combine an MRI scanner with a linear accelerator, allowing acquisition of MRI scans of the patient on each treatment fraction of a radiotherapy (RT) course. On two commercially available systems, acquisition of quantitative MRI was shown to be feasible (1, 2). Daily monitoring of radiotherapy response using quantitative MR imaging biomarkers has become more readily available with the increasing number of MR-linac systems in centers worldwide (3).

Quantitative MRI enables the characterization of tissue properties in a quantitative manner. By measuring this on a daily basis, two exciting ideas for personalized radiotherapy come within reach. The first is to adapt the dose distribution of a treatment plan on a daily basis according to the changing patient biology (4), and the second is to base the total dose that a patient receives on the biological response (5). For this to become clinical practice, the performance of MR-linacs regarding quantitative MRI first needs to be validated (6). Furthermore, it needs to be established if daily changes in imaging biomarkers are detectable and if these changes are associated with clinical outcome.

Perfusion is of interest as it is related to tumor hypoxia, which is a prognostic marker for overall survival in a number of tumor sites (7). An established method for imaging perfusion and permeability in cancer is dynamic contrast-enhanced (DCE) MRI (8). However, as this requires the injection of an MRI contrast agent, DCE MRI is not suitable for daily treatment response monitoring. An alternative to DCE MRI is intravoxel incoherent motion (IVIM) imaging (9), which is a technique based on diffusion-weighted MRI (DWI). In DWI, MR images are sensitized to random motion by the application of strong diffusion-weighting gradients. The amount of diffusion weighting is expressed with the b-value, where a higher b-value indicates stronger diffusion weighting. Typically two or three images are acquired with a different b-value, from which the apparent diffusion coefficient (ADC) is calculated using a mono-exponential model (10). With IVIM, additional low b-values are acquired in order to extract information about perfusion (11). By fitting a bi-exponential model, IVIM allows for the determination of the tissue diffusion coefficient D, the perfusion or blood fraction f, and the pseudo-diffusion coefficient D*, thereby separating perfusion and diffusion effects. In prostate cancer, D (and ADC) parameters were shown to be related to cell density (12, 13). The IVIM parameter f was shown to correlate with blood vessel density in (12).

Changes in IVIM parameters during treatment might provide valuable information about treatment response (4). For cervical cancer, early increases in f have been associated with good response (14, 15). Similarly, in head-and-neck cancer patients, larger reductions in f and higher D values were observed in patients with regional failure compared to patients with regional control (16). In another study with weekly measurements in head-and-neck cancer patients, a significant increase was found in D in complete responders, but no significant differences in f and D* were found between responders and non-responders (17). Daily IVIM measurements in patients with brain metastases showed an increase in D in responders and a decrease in non-responders (18). No significant differences were observed for f and D* between responders and non-responders. For prostate cancer, only DWI has been investigated as a potential biomarker for treatment response. Two studies have shown an increase in the ADC during radiation treatment (19, 20). Therefore, the aim of this multicenter study was to perform daily IVIM measurements in prostate cancer patients to identify if time trends appear in IVIM parameters, which might have potential for treatment response monitoring.

## Materials and Methods

### Patients

Forty-three patients from three institutes with intermediate- and high-risk biopsy-proven prostate cancer were included in this study according to the EAU risk classification (21). Twelve patients were included in the first institute, 8 in the second, and 23 in the third. All patients received the same treatment of 20 fractions of 3 Gy over the course of 4 to 5 weeks on a 1.5 T MR-linac system (Unity, Elekta AB, Sweden). In addition, 34 patients also received androgen deprivation therapy (ADT). Patient demographics are presented in **Table 1**. The study was approved by the institutional review boards and written informed consent was obtained from all patients.

**Table 1:** Patient demographics.

Age	73 (55–83)
iPSA (ng/ml)	8.5 (4.4–37.6)
ADT (months before start of radiation)	2 (0–11)
**ISUP Grade Group**	
1	4
2	19
3	13
4	5
5	2
**T-stage**	
T1a	1
T1c	9
T2a	11
T2b	1
T2c	12
T3a	7
T3b	2

iPSA, initial prostate-specific antigen; ADT, androgen deprivation therapy; ISUP Grade Group, revised prostate cancer grading system introduced by the International Society of Urological Pathology (ISUP). The median (range) is shown for age, iPSA, and ADT.

### MRI

During each treatment fraction, an anatomical T_2_-weighted scan for position verification and an IVIM scan were acquired before the start of irradiation. Thus, the scans on the day of the first fraction provide pre-treatment information. All institutes used the same protocol for the IVIM scan. For the development of the IVIM protocol, previously published guidelines were followed for ADC measurements on the Unity MR-linac (22). A maximum b-value of 500 s/mm^2^ was recommended to compensate for the limited SNR of the Unity MR-linac and to measure at a diffusion time that is comparable to that of diagnostic systems (22). An extra b-value of 30 s/mm^2^ was added to be able to measure IVIM parameters. The averages of the b = 0 s/mm^2^ image were increased to eight. Sequence parameters can be found in **Table 2**.

**Table 2:** Acquisition parameters of the IVIM sequence.

Sequence type	Single-shot echo planar image (ss-EPI)
Field of view (mm^3^)	430 × 430 × 60
Acquired voxel size (mm^3^)	4 × 4 × 4
TR/TE (ms)	2,960/82
b-values (averages) (s/mm^2^)	0 (8), 30 (8), 150 (8), 500 (16)
Gradient timings Δ/δ (ms)	41/20
Fat suppression	SPAIR
SENSE factor	2.3 (left–right)
Phase encoding bandwidth (Hz/pixel)	32.9
Acquisition time (m:ss)	5:11

### Image Registration and Delineation

The T_2_-weighted images of each fraction were registered rigidly to the T_2_-weighted image of the first fraction within a box around the prostate using the correlation ratio as a cost function. This rigid registration allowed for translations and rotations. Next, the b = 0 s/mm^2^ images were registered to the T_2_-weighted image acquired during the same fraction. All registrations were checked visually and improved manually if required.

The prostate and all visible tumors were delineated on the T_2_-weighted image of the first fraction. The tumors were delineated while consulting diagnostic multi-parametric scans acquired according to the PI-RADS v2.1 guideline (23). Tumor delineations were excluded from the prostate delineation to obtain the non-cancerous prostate region. Only the tumor focus with the largest volume was used for the analysis in case of multiple foci per patient. All delineations were propagated to the IVIM scans in order to extract quantitative values. Due to the use of an EPI readout, severe susceptibility artifacts could be present in some IVIM images caused by passing air in the rectum. Therefore, the b = 500 s/mm^2^ images were checked visually and fractions where air was present inside the propagated contours were excluded. The median values of the signal intensities of the voxels inside the resulting delineations were used for calculation of the IVIM parameters.

### IVIM Parameter Calculation

The IVIM parameters were calculated by performing a bi-exponential fit in a segmented fashion to increase robustness (24)

$${\text{S}}_{\text{b}}={\text{S}}_{0}\left(\text{f}{\text{e}}^{-{\text{b D}}^{*}}+\left(1-\text{f}\right){\text{e}}^{-\text{b D}}\right).$$

The diffusion coefficient D was calculated using image intensities at the two highest b-values (150 and 500 s/mm^2^) under the assumption that the contribution of perfusion to the signal at these b-values is negligible (11) using

$$\text{D}=\frac{\ln\left({\text{S}}_{150}/{\text{S}}_{500}\right)}{\left({\text{b}}_{500}-{\text{b}}_{150}\right)}.$$

Here, S_b_ is the signal intensity in the image acquired at a certain b-value. Next, the perfusion fraction f was calculated using the previously calculated D by extrapolating the contribution of the diffusion fraction to S_0_ as follows:

$$\text{f}=1-({\text{S}}_{150}/{\text{S}}_{0}){\text{e}}^{({\text{b}}_{150}\;-\;{\text{b}}_{0})\text{D}}.$$

Finally, D* was calculated using the obtained values of D and f in combination with the signal intensity at the lowest two b-values (0 and 30 s/mm^2^)

$${\text{D}}^{*}=-\frac{1}{{\text{b}}_{30}}\ln\left(\frac{{\text{S}}_{30}/{\text{S}}_{0}-\left(1-\text{f}\right){\text{e}}^{-{\text{b}}_{30}\text{D}}}{\text{f}}\right).$$

### Statistics

To establish if treatment effects could be found on a population level, for each fraction, the mean and the standard error of the mean of the IVIM parameters of all patients were determined for the tumor and non-cancerous prostate. The difference between pre-treatment values of tumor and non-cancerous prostate was tested with a two-sided paired *t*-test with a significance level of α = 0.05.

To determine which changes in IVIM parameters can be attributed to a treatment effect, the repeatability coefficient (RC) of each IVIM parameter was calculated using $\text{RC}=1.96\sqrt{2\;\text{wVar}}$, where wVar is the mean within-patient variance (25, 26). The wVar was determined for the non-cancerous prostate and tumor based on the measurements from the first and second treatment fraction, assuming a negligible influence of the single 3 Gy dose that was received in between. The RC values were related to the size of the ROIs.

To analyze the evolution over time, linear mixed effects analysis was performed using R (v3.6.1) and the lme4 package (27). Separate models were constructed for the D, f, and D* parameters. Fixed effects were fraction (1–20), ROI (non-cancerous prostate/tumor), ISUP group, ADT, and institute. The ISUP scores were divided into a low (ISUP score 1 and 2) and high (ISUP score 3,4, and 5) group. For ADT, the number of months between the start of ADT and start of radiotherapy was used. Patients were included as a random effect. ROIs were modeled as a random effect nested within the patient. This allows the model intercepts to vary among patients and among ROIs within patients. The three models (for D, f, and D*) were constructed separately using backwards elimination as implemented by the step function from the lmerTest package (28). All fixed effects, including their interaction with fraction, were included in the full model. They were then eliminated one at a time based on a significance level of α = 0.05, where the *p*-value was calculated using an *F*-test based on Satterthwaite’s approximation.

## Results

**Figure 1:**
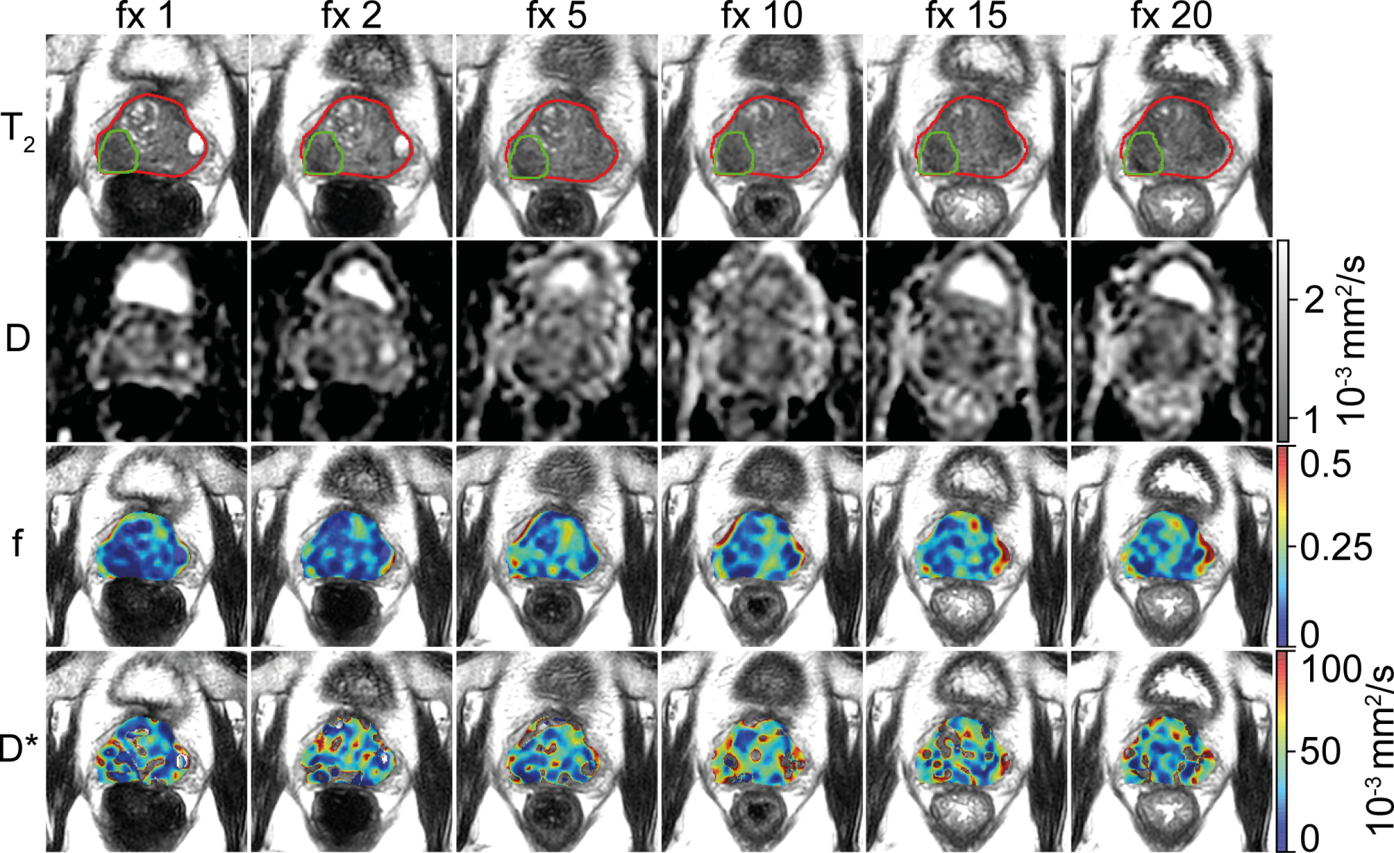
Example of a prostate cancer patient. A voxel-wise map of the IVIM parameters is shown for six treatment fractions (fractions 1, 2, 5, 10, 15, and 20). The prostate is delineated in red, and the tumor is delineated in green. The images are resampled to the reconstructed voxel sizes of the T_2_-weighted acquisition (0.6 × 0.6 × 1.2 mm^3^). Note that during analysis, the voxels from inside the tumor delineation were excluded from the prostate delineation to create the non-cancerous prostate.

For logistical and technical reasons, IVIM scans were missing in 56 out of the total of 860 fractions. Of the remaining 804 fractions, 73 were excluded because of anatomical deformations or susceptibility distortions caused by the EPI readout. Five were excluded because the patient moved between the acquisition of different b-values. This left 726 fractions for analysis with a median number of 18 (range, 9–20) available fractions per patient. In four patients, a tumor could not be distinguished and was not delineated. For those patients, the entire prostate region was analyzed as non-cancerous. **Figure 1** shows the IVIM parameter maps for six fractions from a single patient.

IVIM scans were available for the first fraction in 35 patients. The pre-treatment average and standard error of the mean of D were 1.46 ± 0.02 × 10^−3^ mm^2^/s in the non-cancerous prostate, which was significantly higher (*p* < 0.001) than in the tumor (1.19 ± 0.04 × 10^−3^ mm^2^/s). The pre-treatment average and standard error of the mean of f were 0.086 ± 0.005 in the non-cancerous prostate and 0.088 ± 0.01 in the tumor. The pre-treatment average and standard error of the mean of D* were 28.7 ± 1.4 × 10^−3^ mm^2^/s in the non-cancerous prostate and 28.9 ± 5.4 × 10^−3^ mm^2^/s in the tumor. The pre-treatment values of f and D* were not significantly different between the non-cancerous prostate and tumor.

**Figure 2:**
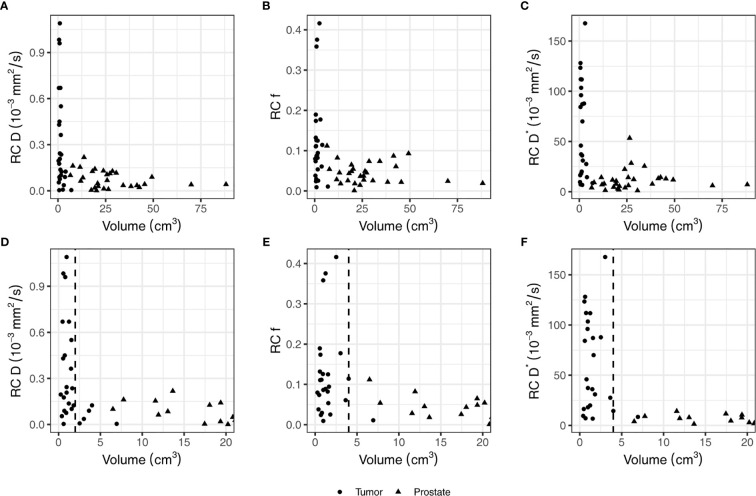
The repeatability coefficients (RCs) of individual patients for the IVIM parameters based on the values from the tumor and non-cancerous prostate on the first and second treatment fraction. Figures **(A–C)** show all data points while figures **(D–F)** show the same data but zommed in on the smaller volumes. In figures **(D–F)**, a vertical dashed line indicates the volume below which the RC steeply increases. This value is 2 cm^3^ in **(D)** and 4 cm^3^ in **(E, F)**.

**Figure 3:**
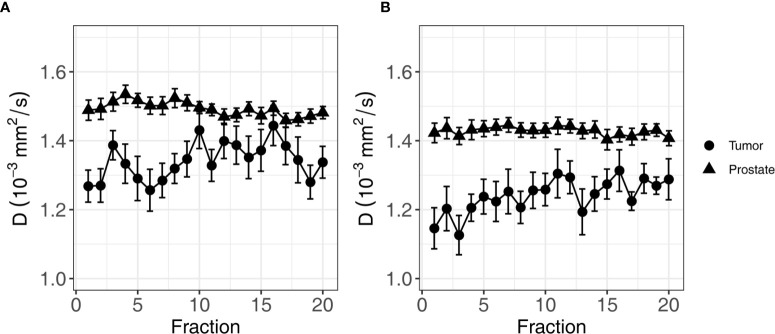
Average of D of all patients over the course of radiotherapy treatment. **(A)** shows the average for patients in the low ISUP group, and **(B)** shows the average for patients in the high ISUP group. Error bars indicate the standard error of the mean. As indicated by the result of the mixed effects model, the increase in the high ISUP group **(B)** is steeper than in the low ISUP group **(A)**.

The RC in the non-cancerous prostate was 0.09 × 10^−3^ mm^2^/s for D, 0.05 for f, and 15.3 × 10^−3^ mm^2^/s for D*. In the tumor, the RCs were 0.44 × 10^−3^ mm^2^/s, 0.16, and 76.4 × 10^−3^ mm^2^/s for D, f, and D*, respectively. **Figure 2** shows that the RC depends on the size of the ROI. The median volume of the non-cancerous prostate delineations was 24 (range, 6.5–88) cm^3^, whereas the median volume of the tumor delineations was 1.0 (range, 0.3–6.9) cm^3^. As shown in **Figure 3D**, the RC of D steeply increases for volumes below 2 cm^3^, and **Figures 2E, F** show a similar increase for f and D* for volumes below 4 cm^3^.

To analyze the evolution over time, mixed effects models were constructed for each IVIM parameter. The set of fixed effects and regression coefficients for each parameter are listed in **Table 3**. For D, these included ISUP groups (low/high, *p*-value = 0.003), the ROI (non-cancerous prostate/tumor, *p*-value < 0.001), the fraction (1-20, *p*-value < 0.001) and interaction terms between ISUP group and fraction (*p*-value = 0.01) and between ROI and fraction (*p*-value < 0.001). **Figure 3** shows the mean of the D for each fraction for the low and high ISUP groups. The effect size for the difference between the group of patients with a high ISUP score compared to patients with a low score was −0.10 ± 0.03 × 10^−3^ mm^2^/s. The D in the non-cancerous prostate was 0.24 ± 0.03 × 10^−3^ mm^2^/s higher than in the tumor. Both ISUP groups and ROI had an interaction term with the fraction number, meaning that the change in D over the course of treatment was different for these groups. In the tumor, for patients with a low ISUP score, the tumor D increased 0.005 ± 0.001 × 10^−3^ mm^2^/s/fraction, whereas for the group with a high ISUP score, the increase was 0.007 ± 0.001 × 10^−3^ mm^2^/s/fraction. This reduces the difference in the D between these groups over the course of treatment: at the 20th fraction, the D as estimated from the model in the tumors of the low ISUP group is increased to 1.38 ± 0.02 × 10^−3^ mm^2^/s and that in the high ISUP group is increased to 1.33 ± 0.03 × 10^−3^ mm^2^/s.

**Table 3:** Model parameters of the mixed effects models for D, f, and D*.

	Regression coefficients (β)	Std. error
**Model for D**	**(10^-3^ mm^2^/s)**	
Intercept (β_0_)	1.284	0.025
Fraction (per one unit)	0.005	0.001
ISUP high (versus ISUP low)	-0.100	0.032
ROI non-cancerous prostate (versus tumor)	0.242	0.026
Interaction Fraction – ISUP high	0.003	0.001
Interaction Fraction – ROI non-cancerous prostate	-0.007	0.001
**Model for f**		
Intercept (β_0_)	0.090	0.007
Fraction (per one unit)	0.002	0.0003
ROI non-cancerous prostate (versus tumor)	0.013	0.006
Institute 1 (versus institute 3)	-0.011	0.010
Institute 2 (versus institute 3)	0.021	0.012
Interaction Fraction – Institute 1	-0.0006	0.0004
Interaction Fraction – Institute 2	-0.001	0.0006
Model for D*	**(10^-3^ mm^2^/s)**	
Intercept (β_0_)	36.6	1.71
Fraction (per one unit)	0.35	0.09
ADT (per one unit)	-1.37	0.35

**Figure 4:**
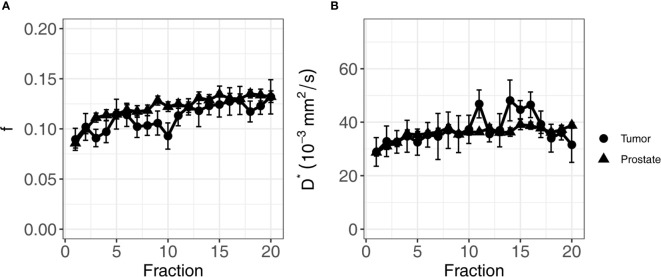
Average of f **(A)** and D* **(B)** of all patients over the course of radiotherapy treatment. Error bars indicate the standard error of the mean. Both f and D* increase over the course of treatment in both the tumor and the non-cancerous prostate.

For the perfusion fraction f, the significant fixed effects were ROI (non-cancerous prostate/tumor, *p*-value = 0.03), the fraction (1-20, *p*-value < 0.001), and the interaction between fraction and institute (*p*-value = 0.04). As institute is part of the interaction term, it was also added to the model as a fixed effect (*p*-value = 0.06) (**Table 3**). The f values in the non-cancerous prostate were 0.013 ± 0.006 higher than in the tumor. An average increase per treatment fraction of 0.002 ± 0.0002 was found in both the non-cancerous prostate and tumor for institutes 1 and 3. For institute 2, this increase was significantly lower (*p*-value = 0.01) at 0.001 ± 0.0005 per treatment fraction, which was the only significant effect containing institute. **Figure 4A** shows the mean f values per fraction grouped by ROI.

For the pseudo-diffusion coefficient D*, the significant fixed effects were the fraction (1-20, *p*-value < 0.001) and ADT (months before the start of treatment, *p*-value < 0.001) (**Table 3**). The D* decreased with -1.37 ± 0.35 mm^2^/s for every month of ADT. The change in D* due to ADT was independent of treatment fraction. The D* increased with 0.35 ± 0.09 × 10^−3^ mm^2^/s each fraction, in both the non-cancerous prostate and the tumor. **Figure 4B** shows the mean values per fraction grouped by ROI.

## Discussion

In this multicenter study, we acquired daily IVIM scans of prostate cancer patients during radiotherapy treatment on three 1.5 T MR-linac systems. IVIM parameters were calculated from the median signal intensities of the tumor and non-cancerous prostate. We analyzed the changes in these parameters over the course of the treatment. The diffusion coefficient D showed an increase in the tumor, while the values in the non-cancerous prostate remained unchanged. The parameters f and D* increased in the tumor as well as in the non-cancerous prostate.

The average pre-treatment D values in the non-cancerous prostate are in line with values reported in the literature, although the range of the reported values in the literature is large: 0.16–1.78 × 10^−3^ mm^2^/s (29). For the tumor, our average pre-treatment D (1.21 ± 0.04 × 10^−3^ mm^2^/s) is higher than previously reported (range 0.13–1.06 × 10^−3^ mm^2^/s) (29). The lower D in the tumor for the high ISUP group is consistent with the literature (29).

The pre-treatment f and D* values in our study are within the range that was previously reported in the literature (29). In their meta-analysis, He et al. found no difference in f between the tumor and the non-cancerous prostate, which is consistent with our pre-treatment findings (29). However, in contrast to our findings, they did find a difference in D* between the non-cancerous prostate and tumor. A reason for this could be the high variance in D* in the current study, in combination with the small standardized mean difference of 0.29 × 10^−3^ mm^2^/s between tumor and non-cancerous prostate reported by He et al. (29).

The RC depended on the size of the ROIs (30, 31). We observed a strong increase in the RC with lower ROI sizes. The mean RC for D in the tumor corresponded to 36% of the mean value in the tumor. This means that a change of 36% would have to occur in order to be significant. While this corresponds to earlier reported values (26), such large changes are not expected in prostate cancer. Van Schie et al. found a change on the group level caused by radiotherapy of 20%, and Foltz et al. found a change of 13% (19, 20). Other tumor sites may have larger tumors, which would reduce their RC, or exhibit larger changes throughout treatment and hold therefore more potential for treatment response monitoring using DWI or IVIM. The same holds for f and D*, where the RCs in the tumor were even higher.

All IVIM parameters, except for D of the non-cancerous prostate, increased during treatment. Interestingly, for the high ISUP group, D increased more during the treatment than for the low ISUP group. This suggests that the cellularity at the end of treatment was similar for both groups. Further work is needed to establish if these observations are linked to treatment outcome. The f and D* also showed an increase during treatment. For D*, we saw an effect of hormonal therapy, where D* was reduced slightly with an increasing duration of ADT before the start of radiation treatment. This is consistent with a reduction in DCE parameters, which is linked to devascularization in patients that received ADT (32, 33). As the entire prostate gland is irradiated, the overall increase in the f and D* values might be caused by an inflammation response of the prostate (34), obscuring more subtle differences that might be present between the tumor and non-cancerous prostate.

A limitation of this study is the use of rigid registration to match the scans from all fractions to the scan of the first fraction. This type of registration cannot account for anatomical deformations caused by, e.g., passing air in the rectum. Moreover, we saw that the contrast of the T_2_-weighted images inside the prostate reduced over the course of treatment, causing the tumor to disappear. This reduction in contrast has been reported before (19, 20, 35), but made it impossible to check the propagated tumor contours visually in later fractions. Because these tumor volumes are relatively small, a small mismatch could lead to a significant difference in the tumor values. In an effort to reduce the influence of small misregistrations, we calculated the IVIM parameters based on the median values of the signal intensities inside the delineations, thereby reducing the effect of outliers.

As indicated by the RCs, the noise in the IVIM acquisition posed problems for voxel-based analysis, especially for the f and D*. This can also be seen in the voxel-wise maps shown in **Figure 1**, where holes appear in the D* maps. This happens when due to noise, the logarithm that is used to calculate D* becomes undefined. By using the median values of the signal intensities for estimation of the parameters, the influence of noise was reduced.

It must be noted that the RC was based on the first two treatment fractions and therefore might include some treatment effect. The RC denotes the smallest significant difference between two measurements taken under identical conditions, with 95% confidence (25). While it is useful for the comparison of the precision of our measurements to previously reported studies, it might not be the right metric to denote a significant change in a time series. As there are multiple measurements per patient, a small change compared to the pre-treatment value that is consistent over time could be statistically significant even if that change is smaller than the RC.

In conclusion, we have successfully acquired daily IVIM scans in prostate cancer patients on the Unity MR-linac system. On a group level, changes in IVIM parameters caused by radiation treatment were found, indicating that it might be useful for treatment response evaluation.

## Data Availability Statement

The raw data supporting the conclusions of this article will be made available by the authors, without undue reservation.

## Ethics Statement

The studies involving human participants were reviewed and approved by medical ethics committee of the Netherlands Cancer Institute; METC19.1644/N18BREL. The patients/participants provided their written informed consent to participate in this study.

## Author Contributions

EK, PH, RK, DT, AT, and UH contributed to the conception and design of the study. VP, MN, FP, AW, A-CM, DT, AT, and UH contributed to the acquisition of data for the study. EK, PH, RK, KS, and UH contributed to the analysis of data for the study. EK and KS performed the statistical analysis. EK wrote the first draft of the manuscript. EK and UH wrote sections of the manuscript. All authors contributed to the article and approved the submitted version.

## Funding

The Netherlands Cancer Institute receives research funding from Elekta. UH receives research funding from ITEA project “STARLIT”. AW and AT acknowledge funding from Cancer Research UK C33589/A28284 and AT also receives support from Cancer Research UK RadNet C7224/A28724. This project represents independent research supported by the National Institute for Health Research (NIHR) Biomedical Research Centre at The Royal Marsden NHS Foundation Trust and the Institute of Cancer Research, London. The views expressed are those of the authors and not necessarily those of the NIHR or the Department of Health and Social Care. The University of Tübingen receives funding from the German Research Council (DFG) grant nos. ZI 736/2-1 and PAK 997/1: MU 4603/1-1 and OT 534/3-1.

## Conflict of Interest

The Netherlands Cancer Institute, the Institute of Cancer Research and Royal Marsden NHS Foundation Trust, and the University of Tübingen are members of the Elekta MR-linac consortium, which aims to coordinate international collaborative research relating to the Elekta Unity (MR-linac). Elekta and Philips are commercial partners within the consortium. Elekta financially supports consortium member institutions with research funding and travel costs for consortium meetings. AT declares research funding from Elekta, Varian, and Accuray. DT declares institutional collaborations including financial and non-financial support with Elekta, Philips, Dr Sennewald, PTW Freiburg, and TheraPanacea. The Department of Radiation Oncology Tübingen (DT and AM) has research collaborations with Elekta AB (Sweden), Philips (The Netherlands), and Siemens Healthineers (Germany).

The remaining authors declare that the research was conducted in the absence of any commercial or financial relationships that could be construed as a potential conflict of interest.

## Publisher’s Note

All claims expressed in this article are solely those of the authors and do not necessarily represent those of their affiliated organizations, or those of the publisher, the editors and the reviewers. Any product that may be evaluated in this article, or claim that may be made by its manufacturer, is not guaranteed or endorsed by the publisher.
